# A Major and Stable QTL for Bacterial Wilt Resistance on Chromosome B02 Identified Using a High-Density SNP-Based Genetic Linkage Map in Cultivated Peanut Yuanza 9102 Derived Population

**DOI:** 10.3389/fgene.2018.00652

**Published:** 2018-12-18

**Authors:** Lifang Wang, Xiaojing Zhou, Xiaoping Ren, Li Huang, Huaiyong Luo, Yuning Chen, Weigang Chen, Nian Liu, Boshou Liao, Yong Lei, Liying Yan, Jinxiong Shen, Huifang Jiang

**Affiliations:** ^1^Key Laboratory of Biology and Genetic Improvement of Oil Crops, Ministry of Agriculture, Oil Crops Research Institute of the Chinese Academy of Agricultural Sciences, Wuhan, China; ^2^College of Plant Science and Technology, Huazhong Agricultural University, Wuhan, China

**Keywords:** peanut cultivars, bacterial wilt resistance, ddRADseq, genetic map, QTLs

## Abstract

Bacterial wilt (BW) is one of the important diseases limiting the production of peanut (*Arachis hypogaea* L.) worldwide. The sufficient precise information on the quantitative trait loci (QTL) for BW resistance is essential for facilitating gene mining and applying in molecular breeding. Cultivar Yuanza 9102 is BW resistant, bred from wide cross between cultivated peanut Baisha 1016 and a wild diploid peanut species *A. chacoense* with BW resistance. In this study, we aim to map the major QTLs related to BW-resistance in Yuanza 9102. A high density SNP-based genetic linkage map was constructed through double-digest restriction-site-associated DNA sequencing (ddRADseq) technique based on Yuanza 9102 derived recombinant inbred lines (RILs) population. The map contained 2,187 SNP markers distributed on 20 linkage groups (LGs) spanning 1566.10 cM, and showed good synteny with AA genome from *A. duranensis* and BB genome from *A. ipaensis*. Phenotypic frequencies of BW resistance among RIL population showed two-peak distribution in four environments. Four QTLs explaining 5.49 to 23.22% phenotypic variance were identified to be all located on chromosome B02. The major QTL, *qBWB02.1* (12.17–23.33% phenotypic variation explained), was detected in three environments showing consistent and stable expression. Furthermore, there was positive additive effect among these major and minor QTLs. The major QTL region was mapped to a region covering 2.3 Mb of the pseudomolecule B02 of *A. ipaensis* which resides in 21 nucleotide-binding site -leucine-rich repeat (NBS-LRR) encoding genes. The result of the major stable QTL (*qBWB02.1*) not only offers good foundation for discovery of BW resistant gene but also provide opportunity for deployment of the QTL in marker-assisted breeding in peanut.

## Introduction

Cultivated peanut (*Arachis hypogaea* L.) is an important oilseed crop in the world (Bertioli et al., 2016; Varshney et al., 2017). The bacterial wilt (BW) caused by *Ralstonia solanacearum* is the most important soil-borne bacterial disease in peanut, especially destructive in China. It usually causes 10–30% yield loss. In the extreme cases, it results in yield loss more than 50% even extinction (Wicker et al., 2007; Yu et al., 2011). This disease is also seriously harmful to tomato, eggplant, chili, potato, tobacco, and other crops (Cao et al., 2009; Liao et al., 2010; Prasath et al., 2011; Narusaka et al., 2014; Xiao et al., 2015). However, the efficacy of chemical control, agricultural cultivation of crop rotation and biological control of BW is very limited, due to the environmental pollution resulted from chemical prevention or the higher cost for rotation and biological control. The most effective and economic measure is to breed and plant BW resistant cultivar (Lyu et al., 2010; Sunkara et al., 2014; Bhatnagar-Mathur et al., 2015).

The quantitative trait loci (QTL) controlling target trait is the important basis for marker-assisted selection (MAS) (Varshney et al., 2005, 2006). Many efforts have been made to identify molecular markers linked to the BW resistance for molecular breeding in peanut (Jiang et al., 2007; Ren et al., 2008; Peng et al., 2010; Hong et al., 2011; Zhao et al., 2016). Ren et al. (2008) identified two markers linked to BW resistance with genetic distance of 8.12 and 11.46 cM using 45 polymorphic amplified fragment length polymorphism (AFLP) markers and bulk segregant analysis (BSA) analysis. Peng et al. (2010) detected three QTLs for BW resistance based on a linkage map consisting of 98 AFLP markers and covering 285 cM map length. The analysis of these studies was based on the linkage maps with insufficient markers that linked with the resistant trait, thus limiting the use of MAS in the BW resistance breeding. Therefore, it is necessary to construct dense genetic map for precise QTL location.

Furthermore, identifying and pyramiding of QTLs derived from different resistant source is helpful to improve the resistant level and stability in BW resistance cultivar (Shi et al., 2008). Recently, two major QTLs based on a Yueyou 92 derived population was reported (Zhao et al., 2016). The source of resistance in Yueyou 92 can be traced back to Xiekangqing, a cultivated peanut with high level BW resistance. Meanwhile, Yuanza 9102, another popular cultivated peanut, has obvious resistance to BW in different condition too. However, the BW resistance of Yuanza 9102 is contributed by its parental line *A. chacoense*, a diploid wild peanut which has rich resistant resource. Whether the genetic basis of BW resistance in Yuanza 9102 and Yueyou 92 are same is unclear. It is worthy of understanding the genetic basis of BW resistance in Yuanza 9102 through QTL mapping.

With the development of the next generation sequencing (NGS), the high-throughput technology provides a convenient and quick platform for calling SNP markers and genotyping on a large scale (Baird et al., 2008; Peterson et al., 2012). The double-digest restriction-site-associated DNA sequencing (ddRADseq) based on the next-generation sequencing has many advantages, such as reduced genomic complexity, high effectiveness, and moderate-cost (Baird et al., 2008; Davey et al., 2011, 2013; Peterson et al., 2012; Gupta et al., 2015). Therefore, it has a wide range of applications on high-resolution genetic map, genome sequencing assembly as well as genetic analysis of complex genomic research (Peterson et al., 2012; Chen et al., 2013; Recknagel et al., 2013).

Molecular MAS provides an efficient tool for breeding as it offers rapid and precise selection of the targeted trait. In peanut, the technology has been used to improve several traits, such as rust resistance, nematode resistance, and the high oleic acid (Varshney et al., 2010, 2014a; Chu et al., 2011; Sukruth et al., 2015; Janila et al., 2016). However, the previous method for identification BW resistance mainly relies on phenotypic investigation in peanut field. Since the degree of the BW disease can be affected by many factors, such as soil conditions, bacteria concentration, rainfall and temperature, phenotypic investigation needs to be repeated for many different conditions. It is necessary to establish a quick, simple, and convenient method to improve the efficiency of peanut BW resistance selection. Combing precise QTL and closely linked molecular markers, resistant materials can be identified in the early generations using MAS technique. This will greatly improve the efficiency of peanut BW resistance breeding.

In this study, we employed ddRAD-seq approach to identify large scale genome-wide SNPs using the recombinant inbred line (RIL) population of Yuanza 9102 × Xuzhou 68-4. A SNP-based genetic linkage map was constructed and the syntenic analysis with AA and BB genome of diploid wild peanut was performed. Moreover, we identified QTLs for BW resistance in peanut and predicted candidate BW resistance genes for further investigation.

## Materials and Methods

### Plant Materials

A RIL population including 188 F_7_ lines derived from the cross between Yuanza 9102 and Xuzhou 68-4 was used in this study. Yuanza 9102 is a popular cultivar in China that is resistant to *R. solanacearum*, while Xuzhou 68-4 is susceptible *to R. solanacearum*. Yuanza 9102 had wild-derived source of BW resistance which was derived from the cross of Baisha 1016 × *A. chacoense* (Xiong et al., 2003). The RIL lines were grown using randomized complete block design with three replications at two experimental locations. One was at experimental station of Nanchong (NC), Sichuan, China and the other was at experimental station of Hongan (HA), Hubei, China. Each accession was planted in three rows. Resistant parent Yuanza 9102 and susceptible parent Xuzhou 68-4 were planted after every 50 accessions as resistant and susceptible controls. There were 10 plants in each row with plant-to-plant distance of 10 cm. The row-to-row space was 30 cm apart. Genomic DNA was extracted from young leaf tissue essentially as described by Grattapaglia and Sederoff (1994).

### ddRAD Library Construction and Sequencing

Genomic DNA was digested with *EcoR*I and *Mse*I at 37°C for 6 h and then heat inactivated at 65°C for 90 min. The ligation with barcode adapter and common adapter, purification of pooled DNA template with specific adapters, and PCR enrichment were performed using a protocol described by Chen et al. (2013). Fragments of 250–450 bp were collected and dissolved in elution buffer for sequencing. The ddRAD library was sequenced on a Hiseq4000 next-generation sequencing platform. Raw Illumina reads with poor quality, contaminant sequences were filtered out using NGS QC Toolkit and data of individuals were separated according to barcode. The sequences of barcode were then removed and the clean data of each accession were used for subsequent analysis.

### SNP Discovery and Genotyping

Using stacks-0.9998 software (Catchen et al., 2011, 2013), the reads of the specific locus *EcoR*I and *Mse*I flanking genomic DNA fragment were proceeded to cluster analysis and SNPs detection. The parameter of parents was set as: stacks-0.9998/ustacks -t gzfastq -r -d -m 5-M 2 -p 15, and the parameter of offspring was set as: stacks-0.9998/ustacks -t gzfastq -r -d -m 3 -M 2 -p 15. Using cstack software, all the loci of two parents were clustered as a catalog. The parameters was set as: stacks-0.9998/cstacks -b 1 -s QT0859 -s QT0860 -p 15 -n 3. Using sstack software, the stacks of RIL individuals were aligned to the catalog of parents and the parental alleles were determined in the progeny. The parameter was set as: stacks-0.99998/sstacks -p 15 -b 1 -c ^∗^/result/batch_1 -s ^∗^/result/sample.fq -o ^∗^/result. Only the homozygous and polymorphic SNP markers were chosen for following analysis. In order to use the data more effectively, probabilistic PCA (PP) method was chosen to supplement the genotype data of each line (Fu, 2014). This method is based on PCA algorithm and has rapid computing speed and higher accuracy. Then, the proportion of missing data should be no more than 30%. The sequences of obtained SNPs were aligned against AA and BB genome of wild peanut *A. duranensis* and *A. ipaensis* (Bertioli et al., 2016), and only the sequences that uniquely mapped the genomes with the similarity and coverage over 95% were chosen. The remained SNPs were used for map construction. The developed SNP markers were designated as AHES (*Arachis hypogea* EcoRI-digestion genomic SNP) and AHMS (*Arachis hypogea* MseI-digestion genomic SNP) as described in Shirasawa et al. (2012).

### Genetic Map Construction and Synteny Analysis

The genotypes of the 188 RIL F_7_ individuals were used to construct a genetic map. LG assignments and marker order were based on the regression mapping of Joinmap 4.0 software (Van Ooijen, 2006). The map length was estimated using Haldane’s mapping function. The graphic of the genetic map was represented using Mapchart 2.0 software (Voorrips, 2002). Markers were tested for segregation distortion by the chi-square test. The tags including the SNP markers of the genetic map were aligned with AA and BB genomes using blat software. The syntenic relationship was determined according to the physical location and genetic map distance and the graphic was displayed with circos figure.

### Evaluation of Resistance to Bacterial Wilt

All individuals of the RIL population and their parents were planted in the BW disease nursery in Hongan and Nanchong. These disease nurseries were specially used for the evaluation of resistance to BW caused by *R. solanacearum* since 2007. The phenotype of BW resistance was investigated at Hongan in 2014 and 2015 and at Nanchong in 2015 and 2016. The method of evaluation of BW resistance was according to that described previously by Jiang et al. (2006) with small modification. At the seedling stage, flowering-pegging stage and harvest stage, the survival rate of each accession were investigated. Survival rate of each accession equals healthy plants/total plants × 100%. The resistance to BW was evaluated using the following standard: resistant, survival rate was of >80%; moderately resistant, survival rate ranged from 65 to 80%; moderate susceptible, survival rate ranged from 35 to 65%; susceptible, survival rate was <35%.

### Statistical Analysis of Phenotyping Data

The broad-sense heritability was calculated as *h^2^ = σ^2^_g_/(σ^2^_g_+ σ^2^_ge_ / n + σ^2^_e_/nr)* according to Hallauer and Miranda’s method (Hallauer and Miranda, 1998). In the formula, *σ^2^_g_* represents the genetic variance, *σ^2^_ge_* represents the interaction variance of the genotype with environment, σ^2^_e_ represents the variance of residual error, *n* represents the number of environments and *r* represents the number of replications. The calculation of the variance components was obtained using the SAS software by general linear model (GLM) procedure.

### QTL Mapping for BW Resistance

Quantitative trait loci analyses were performed using the software WinQTLCart 2.5 (Wang S. et al., 2012) and program of composite interval mapping (CIM). The CIM analysis was performed using Model 6 and forward regression method. The number of control markers, window size, and walk speed were set as 5, 10, and 2 cM, respectively. The threshold of LOD for declaring the presence of a QTL was determined by 1000 permutation tests at a significance level of *P* < 0.01. QTLs had phenotypic variation explained more than 10% were considered as major QTLs. QTLs detected in different year/season were designated as consistent QTLs. The same QTL appeared in different locations were refereed as stable QTLs (Varshney et al., 2014b). QTLs were designated with an initial letter ‘q’ followed by the trait name and the LG corresponding chromosome, according to the previous described nomenclature (McCouch et al., 1997). If more than one QTL was detected for the same trait and linkage group (LG), a number was added after the LG. A graphic representation of the QTL was generated using Mapchart 2.0 software (Voorrips, 2002).

## Results

### ddRAD Sequencing and Identification of SNP Markers

**FIGURE 1:**
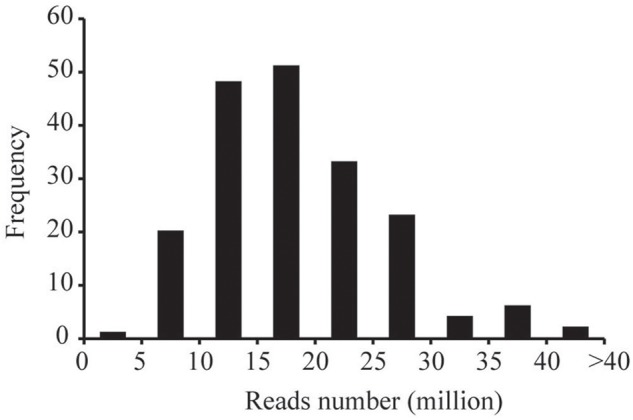
The frequency of the numbers of sequencing reads for the RIL individuals.

The ddRADseq protocol was used to construct reduced representation libraries for the parents (Yuanza 9102 and Xuzhou 68-4) and their 188 RIL lines. Genomic DNA was double digested separately with restriction enzymes *Eco*R I (GAATTC) and *Mse* I (TTAA). Size of insert fragments in the libraries varied from 160 to 360 bp. The ddRAD-seq libraries were sequenced on the Illumina HiSeq4000 platform. In total, ∼361.03 Gb clean data (Q20>96%) were generated, containing 3719.77 million reads, with each read being ∼90 bp in length. Among these data, 161.39 million reads came from the parents, and ∼3558.38 million reads came from the libraries for the 188 RIL lines. The reads numbers of RIL individuals were 4.99–40.68 million range, with an average of 18.93 million reads (Figure 1).

After ustacks clustering analysis and SNP detection of all clean reads, 9,688 and 20,728 SNPs were identified from the reads of the specific locus *EcoR*I and *Mse*I flanking genomic DNA fragments respectively. Among these, a total of 18,494 homozygous and polymorphic SNP markers were detected. The obtained stacks of each RIL lines were aligned against the catalog of all loci of two parents to determine their genotype at each locus. To avoid interference of paralog and repetitive sequence, the consensus sequence of the obtained SNPs were blasted against AA and BB genome of wild peanut and only the uniquely mapped reads were retained. Finally, 2,324 makers in the genome were identified for map construction.

### Construction of High-Resolution Genetic Map and Comparative Analysis

**FIGURE 2:**
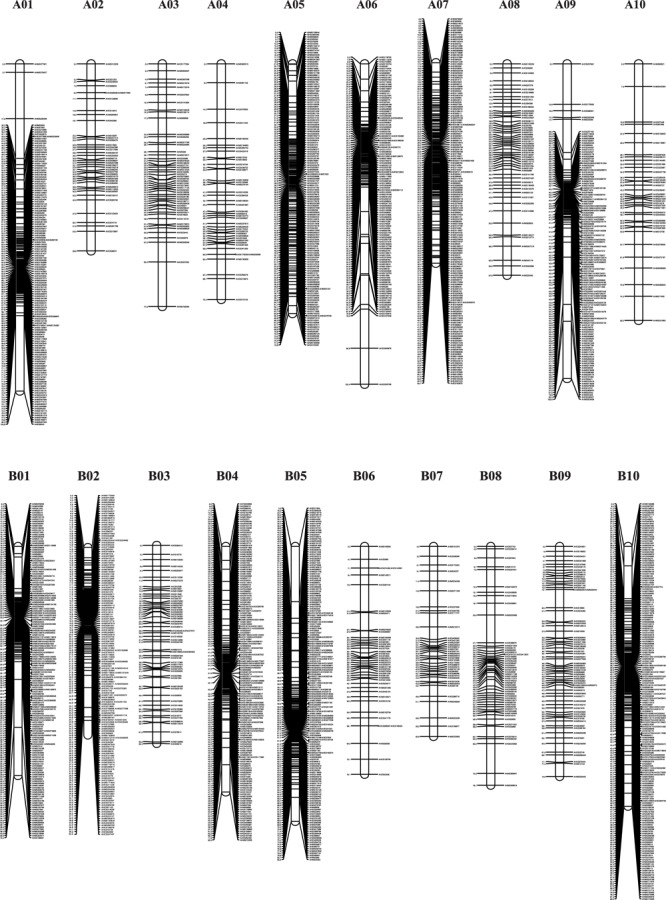
Graphical presentation of the SNP-based genetic linkage map constructed in the RIL population derived from a cross between Yuanza 9102 and Xuzhou 68-4. Marker names are shown on the right side of each chromosome, with map distances (in cM) shown on the left.

**FIGURE 3:**
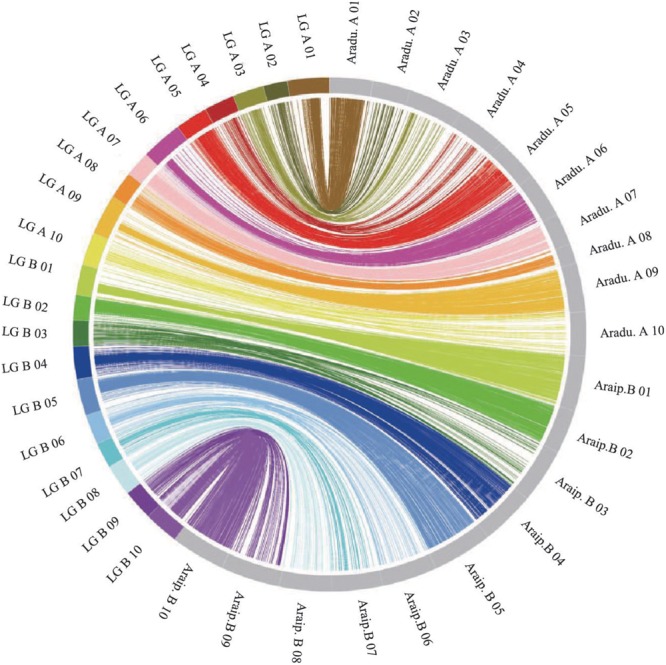
Circos diagram representing syntenic relationships between genetic linkage map in this study and AA and BB genomes of wild peanut. Each color represents a match between the linkage group (LG) of the genetic map and the corresponding chromosome of AA/BB genome.

The genotypes of the 188 RIL F_7_ individuals were used to construct the genetic map using Joinmap 4.0 software. Overall, a genetic map was constructed, which contained 2,187 SNPs and 1,962 mapped loci distributed into 20 LGs, representing 20 chromosomes, with a map length of 1,566.1 cM (Figure 2 and Table 1). The SNP marker sequences on the genetic map were listed in Supplementary Table S1. The LGs ranged from 59.8 to 104.6 cM in length with an average of 78.30 cM, and 10 LGs contained over 100 markers. A02 is the shortest LG spanning 59.8 cM and contains only 40 markers, whereas A01 is the longest LG spanning 104.6 cM and contains 141 markers. The marker densities ranged from 0.37 to 2.22 cM/locus, resulting in an average distance of 0.72 cM between markers for the entire map (Figure 2 and Table 1). The Chi square analysis identified 987 SNPs (45.1%) with segregation distortion. All 20 LGs had good syntenic relationship with correspondence to AA genome and BB genome of diploid wild peanut (Figure 3).

**Table 1:** Characteristics of the SNP-based genetic linkage map constructed in this study.

LGs	Length	No. of	No. of	Density
	(cM)	markers	loci	(cM/locus)
A01	104.6	141	137	0.74
A02	59.8	40	39	1.5
A03	77.6	51	51	1.52
A04	75.3	41	40	1.84
A05	79.8	143	140	0.56
A06	102.4	125	118	0.82
A07	64.1	167	163	0.38
A08	67.6	54	54	1.25
A09	100.6	164	125	0.61
A10	82	37	37	2.22
B01	75.5	202	154	0.37
B02	62.2	167	153	0.37
B03	65.3	57	55	1.15
B04	80.9	192	157	0.42
B05	90.6	197	162	0.46
B06	75.1	42	41	1.79
B07	62.6	37	37	1.69
B08	78.7	53	52	1.48
B09	75.8	67	65	1.13
B10	85.6	210	182	0.41
Total	1566.1	2187	1962	0.72

### Phenotypic Variation of Bacterial Wilt Resistance

**FIGURE 4:**
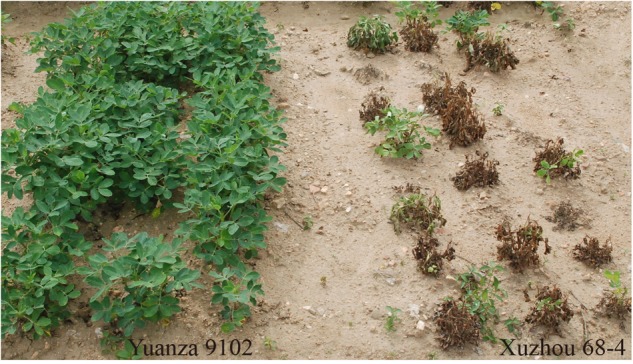
Phenotypes of resistant and susceptible parents for BW disease. Left three lines were Yuanza 9102, which is the BW-resistant parent. Right three lines were Xuzhou68-4, which is the BW-susceptible parent.

**FIGURE 5:**
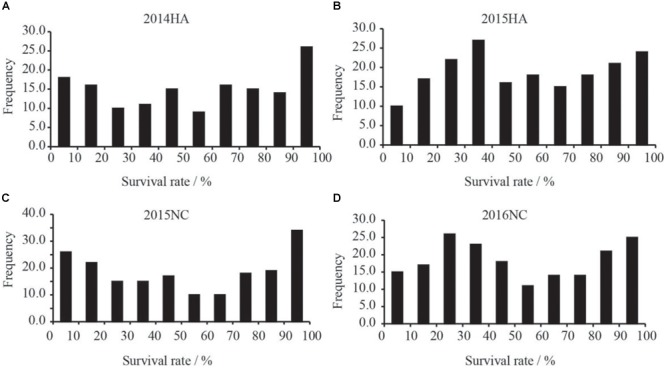
Frequency distribution xof survival rates of RILs population in BW disease nursery. X-axis indicate the range of survival rates of peanut. **(A)** Distribution of survival rate of the RILs grown in Hongan in year 2014. **(B)** Distribution of survival rate of the RILs grown in Hongan in year 2015. **(C)** Distribution of survival rate of the RILs grown in Nanchong in year 2015. **(D)** Distribution of survival rate of the RILs grown in Nanchong in year 2016.

The survival rate of parents and their RIL lines were investigated in disease nursery under four different environment conditions (2014HA, 2015HA, 2015NC, and 2016NC). The survival rate of the resistant parent Yuanza 9102, was significantly higher than that of the susceptible parent Xuzhou 68-4 (Figure 4 and Table 2). Frequency distribution showed that BW resistances had good consistency in four environment conditions (Figure 5 and Table 2). Obvious phenotypic variations of resistance to BW were observed among RILs population under all four environment conditions. The survival rates of individual lines ranged from 0 to 100% and displayed two-peak distribution in four environment conditions (Figure 5). These results suggest that the resistance to BW is controlled by a major effect gene and a series of minor effect genes. ANOVA of BW trait across all four environment conditions indicated that genotype (G), environment (E) and genotype–environment interactions (G × E) had significant effects on the trait (Table 3). Moreover, the trait of BW resistance showed high broad-sense heritability of 81.72% (Table 3). These results suggest that genetic factor plays a major role in the expression of BW resistance.

**Table 2:** Descriptive statistical analysis of BW resistance.

Env.	Yuanza 9102 (%)	Xuzhou 68-4 (%)	Population					
			Mean (%)	*SD*	Min (%)	Max (%)	Skew	Kurt
2014HA	90.91	25	53.78	32.68	0	100	–0.15	–1.30
2015HA	77.82	16.67	52.72	28.30	0	100	0.05	–1.26
2015NC	77.27	16.67	52.64	34.58	0	100	–0.05	–1.40
2016NC	65.28	9.13	50.83	29.94	0	100	0.12	–1.32

*Env., environments; 2014HA and 2015HA, the phenotype of BW was investigated at experimental stations of Hongan, Hubei Province, in year 2014 and 2015; 2015NC and 2016 NC, the phenotype of BW was investigated at experimental stations of Nanchong, Sichuan Province in year 2015 and 2016.*

**Table 3:** Variance (ANOVA) Analysis and the broad-sense heritability (*h^2^*) for BW resistance of RIL lines in multi-environments.

Source	*df*	SS	MS	*F*-value	*P*	*h^2^* (%)
Genotype	187	579112.03	3096.86	11.19	<0.0001	0.82
Environment	3	1312.39	437.47	1.58	<0.0001	
Genotype × Environment	518	293294.15	566.21	2.05	<0.0001	
Error	183	50625.05	276.64			

*SS, sum of square; MS, mean square; *df*, degrees of freedom; *h*^2^, the broad-sense heritability.*

### Detection of QTLs for Bacterial Wilt Resistance

**FIGURE 6:**
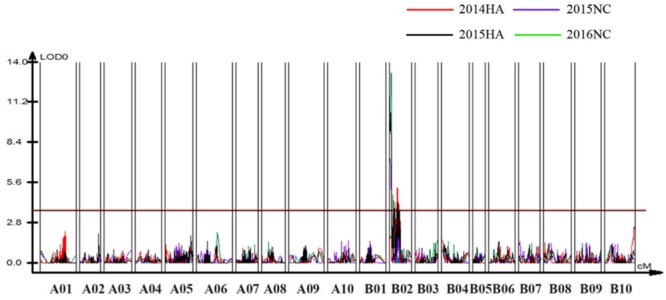
Quantitative trait loci (QTLs) for BW resistance of Yuanza 9102 × Xuzhou 68-4 population. The horizontal line represents a LG-wise logarithm of odds (LODs) significance threshold.

**FIGURE 7:**
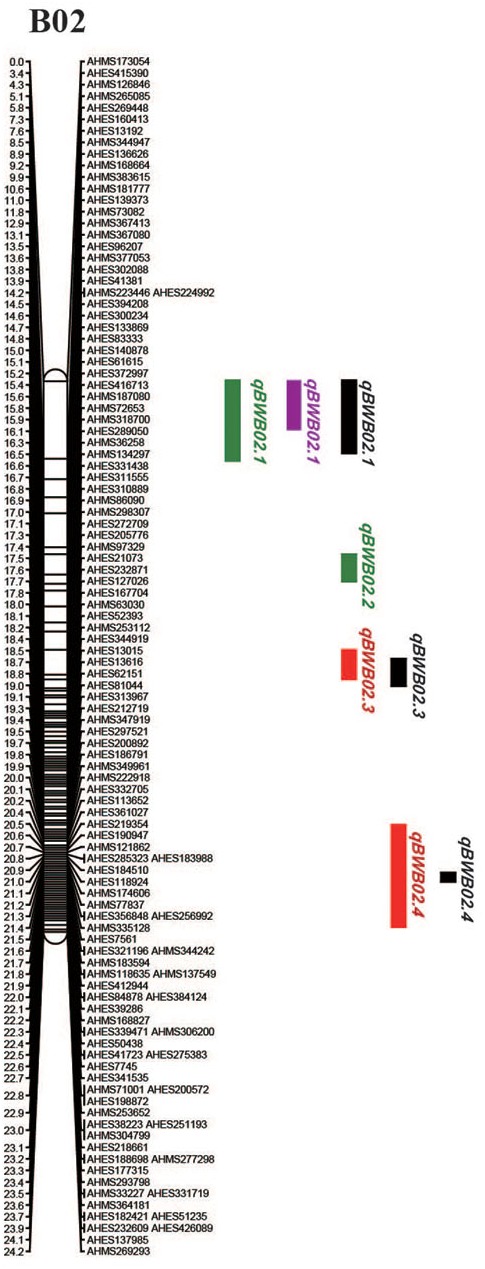
Positions of the QTLs on the B02 of the genetic linkage map. Scale bars on the left side describe the map distance in cM. The QTLs in 2014HA, 2015HA, 2015NC, 2016NC were shown as red, black, purple, and green, respectively.

To identify QTLs for BW resistance, phenotypic data of four environment conditions were analyzed together with the genetic mapping data of 2,187 markers. A total of four significant QTLs were identified through single-environment QTL analysis. The four QTLs were all distributed on chromosome B02 and could explained 7.72–23.33% phenotypic variation of resistance to BW (Figures 6, 7 and Table 4). Among them, the stable QTL *qBWB02.1* was repeatedly detected in three environment conditions of 2015NC, 2016NC, and 2015HA (Figures 6, 7 and Table 4). The major stable QTL (*qBWB02.1*) could explain phenotype variation of 12.17–23.33%, and the highest PVE (23.33%) with LOD of 13.29 was in 2016NC environment. The *qBWB02.1* was located at 0–3.5 cM map position on chromosome B02 with flanking markers AHMS173054 and AHES415390 (Figures 6, 7 and Table 4). This region could be traced to the genomic region of B02 of *A. ipaensis*. Corresponding position of 3.5 cM length on the genetic map was about 2.3 Mb in the physical map, i.e., 2,501,128 to 4,797,039 bp. In the RIL lines of the extreme separation of BW resistance, 21 lines (88% resistant lines) with the flanking markers of *qBWB02.1* region show stable resistant to BW (survival rate >80%) and thirty lines (86% susceptible lines) without the flanking markers show stable susceptible to BW (survival rate <35%, Supplementary Table S2). The consistent QTLs *qBWB02.3* and *qBWB02.4* were both repeatedly detected in 2014HA and 2015HA. In addition, BW resistance alleles at all QTLs were contributed by Yuanza 9102.

**Table 4:** Quantitative trait loci (QTL) analysis for BW resistance in RILs population.

QTL	Chr.	Marker interval	Range (cM)	Env.	Position	Additive	PVE (%)	LOD
*qBWB02.1*	B02	AHMS173054-AHES415390	0–3.5	2016NC	2.01	16.07	23.33	13.29
				2015NC	0.01	13.56	12.17	7.29
				2015HA	2.01	13.30	16.31	10.50
*qBWB02.2*	B02	AHES13192-AHES136626	7.6–8.8	2016NC	7.61	12.72	9.57	4.78
*qBWB02.3*	B02	AHMS73082-AHES96207	11.8–13.4	2014HA	12.91	13.31	9.33	4.24
				2015HA	12.91	10.72	7.72	3.83
*qBWB02.4*	B02	AHES297521-AHES426089	19.5–24	2014HA	21.84	10.99	8.03	4.48
				2015HA	21.91	7.53	5.49	4.26

*Chr., chromosome; PVE, phenotypic variance explained by each QTL; Env., environment; 2014HA, 2015HA, 2015NC, and 2016NC, Hongan in 2014, Hongan in 2015, Nanchong in 2015, Nanchong in 2016, respectively.*

### Prediction of Candidate Resistant Genes on Chromosome B02

To identify the candidate genes for BW resistance in the stable major QTL loci (*qBWB02.1*), the target region (AHMS173054 to AHES415390) was examined for predicted genes according to *A. ipaensis* reference genome annotation database at Peanutbase^1^ and the analysis of nucleotide-binding site -leucine-rich repeat (NBS-LRR)-encoding genes of *A. ipaensis* reference genome by Bertioli et al. (2016). The region of *qBWB02.1* spanning 3.5 cM linkage interval corresponds to ∼2.3 Mb genomic region of B02 (physical position B02: 2,501,128–4,797,039 bp) of *A. ipaensis*. A total of 154 annotated genes were identified in this region. Of which, 21 genes (Table 5) were found to have disease resistance protein function. All these genes belonged to NBS-LRR genes, which was the largest class of resistance (*R*) genes. The 21 resistance proteins contained NBS domain and without Toll-interleukin-1 receptor (TIR) domain. Leucine-rich repeat (LRR) domain was also included in all proteins, except Araip.8GZ6F (Table 5). Our data suggests that the major QTL *qBWB02.1*, which was consistent and stable in three environment conditions, was a resistant genes-dense region. Therefore, these 21 genes harboring this genome segments provide potential disease resistant genes for BW resistance and warrant further investigation.

**Table 5:** The predicted candidate genes for BW resistance in the major stale QTL (*qBWB02.1*) covering segments of pseudomolecule.

Gene model	Chr.	Gene location	Class	TIR	NBS	LRR	Annotation
Araip.2M16Z	Araip.B02	2478930–2505968	NBS-LRR	x	NBS	LRR	Disease resistance protein
Araip.EW22T	Araip.B02	2506040–2520876	NBS-LRR	x	NBS	LRR	Disease resistance protein
Araip.RB9A1	Araip.B02	2520900–2544746	NBS-LRR	x	NBS	LRR	Disease resistance protein
Araip.654N3	Araip.B02	2544782–2556564	NBS-LRR	x	NBS	LRR	Disease resistance protein
Araip.S2E2N	Araip.B02	2572866–2579719	NBS-LRR	x	NBS	LRR	Disease resistance protein
Araip.M9Z94	Araip.B02	2596074–2599895	NBS-LRR	x	NBS	LRR	Disease resistance protein
Araip.8GZ6F	Araip.B02	2733170–2734382	NBS-LRR	x	NBS	X	Disease resistance protein
Araip.U522J	Araip.B02	3705209–3707314	NBS-LRR	x	NBS	LRR	Disease resistance protein
Araip.G1MIP	Araip.B02	3738963–3743112	NBS-LRR	x	NBS	LRR	Disease resistance protein
Araip.VE4DY	Araip.B02	3919279–3923238	NBS-LRR	x	NBS	LRR	Disease resistance protein
Araip.05JB8	Araip.B02	3937798–3941473	NBS-LRR	x	NBS	LRR	Disease resistance protein
Araip.JMY44	Araip.B02	3957980–3961994	NBS-LRR	x	NBS	LRR	Disease resistance protein
Araip.DP41N	Araip.B02	3970971–3973637	NBS-LRR	x	NBS	LRR	Disease resistance protein
Araip.BR51T	Araip.B02	3977332–3982425	NBS-LRR	x	NBS	LRR	Disease resistance protein
Araip.AG0F8	Araip.B02	3996300–4002038	NBS-LRR	x	NBS	LRR	Disease resistance protein
Araip.AH01G	Araip.B02	4032807–4045456	NBS-LRR	x	NBS	LRR	Disease resistance protein
Araip.YCN52	Araip.B02	4066553–4069770	NBS-LRR	x	NBS	LRR	Disease resistance protein
Araip.WHQ43	Araip.B02	4076909–4079481	NBS-LRR	x	NBS	LRR	Disease resistance protein
Araip.U0YME	Araip.B02	4108921–4111605	NBS-LRR	x	NBS	LRR	Disease resistance protein
Araip.WF303	Araip.B02	4119524–4128447	NBS-LRR	x	NBS	LRR	Disease resistance protein
Araip.65IVT	Araip.B02	4130016–4138256	NBS-LRR	x	NBS	LRR	Disease resistance protein

*TIR, toll-interleukin-1 receptor domain; NBS, nucleotide-binding site domain; LRR, leucine-rich repeat domain; TIR/NBS/LRR, presence/absence of TIR/NBS/LRR domain.*

## Discussion

Cultivated peanut (*A. hypogaea* L.) is an allotetraploid (AABB, 2n = 4 × = 40) species with a large genome and low genetic diversity within the species. Because of these features, the construction of genetic linkage map in cultivated peanut has always been a formidable task. In recent years, many efforts have been made to construct genetic maps (Halward et al., 1993; Burow et al., 2001; Herselman et al., 2004; Foncéka et al., 2009; Varshney et al., 2009; Hong et al., 2010; Gautami et al., 2012; Qin et al., 2012; Shirasawa et al., 2012, 2013; Sujay et al., 2012; Wang H. et al., 2012; Seck et al., 2013). Varshney et al. (2009) constructed a genetic linkage map containing 135 SSR markers distributed on 22 LGs. Guo et al. (2012) developed a map covering 1,278 cM based on 449 SSR markers. Moreover, Shirasawa et al. (2012) generated a map covering 2,166.4 cM with 1,114 loci using SSR and transponson markers. However, these maps were constructed using low throughput molecular markers with low density, thus unable to provide precise information on the QTLs controlling the traits of interest. In addition, the development of these polymorphic markers requires more screening work, cost for primer synthesis as well as resources and time. More efficient type of marker and a high amount of markers are crucial for improving the density and quality of linkage maps in peanut.

SNPs are particularly attractive for genetic map construction, as they represent the most common type of DNA polymorphism in the genome and are amenable to high-through genotyping. Currently, there are a few studies which develop the SNP markers in peanut using the method of reduced genome sequencing, chips and resequencing (Nagy et al., 2012; Zhou et al., 2014; Shasidhar et al., 2017; Agarwal et al., 2018). Using ddRAD-seq method, Zhou et al. (2014) constructed the first high density SNP-based linkage map in tetraploid peanut consisting of 1,685 markers, 1267 loci, spanning a distance of 1,446.7 cM. Using resequencing method, Agarwal et al. (2018) developed the densest genetic map currently available for cultivated peanut with 8,869 SNPs and 2,156 mapped loci. In our SNP-based genetic map, 2,187 SNPs (1,962 loci) were identified using ddRAD-seq and were assigned to 20 LGs corresponding to 20 chromosomes of cultivated peanut. The total length of the genetic linkage map was 1,566.1 cM, with an average inter-loci distance of 0.72 cM. The genetic map constructed in this study is highly cost-effective. On the other hand, the linkage map constructed in this study used a RIL F_7_ generation as material. In general, the markers were more likely skewed segregation in high generation population of RIL population. This phenomenon probably related to gametophyte and/or zygotic natural selection and chromosomal rearrangements in many generations and artificial sampling involved in the construction of the RIL population. In fact, 987 (45.1%) markers of this genetic map showed skewed segregation. While the high generation population could improve the accuracy of bioinformatics analysis for SNP discovery. On the whole, the big loci number of the genetic map and its good synteny with AA and BB genomes provide a good foundation for important traits QTL mapping in peanut.

Based on this high-density genetic map, we detected four QTLs associated with BW resistance, including a major QTL (*qBWB02.1*) and three minor QTLs (*qBWB02.2, qBWB02.3, qBWB02.4*). *qBWB02.1* was detected in both locations (Nanchong and Hongan) and 2 years (2015, 2016) and was considered to be consistent and stable. *qBWB02.3* and *qBWB02.4* were detected in 2 years (2014, 2015) and were considered to be consistent. Comparing the QTLs for BW resistance identified with previous studies (Jiang et al., 2007; Ren et al., 2008; Zhao et al., 2016), the QTLs located in our study were shown to be novel QTLs for BW resistance and the QTL interval in our study was significantly smaller than previously studies.

All four QTLs had positive additive effects, indicating these alleles were derived from resistant parent Yuanza 9102. From the pedigree of Yuanza 9102, we knew that the BW resistance was introgressed from diploid wild species of *A. chacoense. A. chacoense* is a good resistance resource of BW and some high BW-resistance accessions were obtained from this species (Chen et al., 2008). On the other hand, some different resistant alleles in QTLs detected by Zhao were derived from Yueyou 92 (Zhao et al., 2016). The source of resistance of Yueyou 92 can be traced back to Xiekangqing, a famous resistant cultivated peanut in China. In addition, previous study showed that the resistant gene of Yuanza 9102 exhibited dominance or partial dominance effects (Ren et al., 2008), while the resistant gene of Yueyou 92 was recessive (Zhao et al., 2016). The different QTL results may come from the different materials used.

In the present study, 21 disease resistance genes (Table 5) in the region of major QTL *qBWB02.1* were identified. We considered these genes as putative candidate genes for BW resistance. All proteins encoded by the 21 genes contained the necessary domains (NBS and LRR) of NBS-LRR class. The domain can induce a programmed cell death or hypersensitive response (HR) in the presence of a specific pathogen elicitor (Bonardi et al., 2011). Although several QTLs related to resistance to BW have been identified in tomato (Wang et al., 2000; Carmeille et al., 2006), tobacco (Qian et al., 2013), *Arabidopsis thaliana* (Godiard et al., 2003), *Medicago truncatula* (Ben et al., 2013), very few resistance genes have been identified except for *ERECTA* gene and *RRS1*-R gene of *A. thaliana*. *RRS1*-R gene in *A. thaliana*, which is involved in monogenic resistance to BW, contains typical TIR-NB-LRR domain. It recognizes avirulence gene *PopP2* and traffics to the nucleus through nuclear localization signal (Deslandes et al., 2002, 2003). Another R gene *ERECTA* in *A. thaliana*, as a quantitative resistance locus, involves in polygenic resistance to BW. In addition, overexpression of peanut NBS-LRR gene *AhRRS5* in tobacco significantly enhanced the resistance to BW (Zhang et al., 2017). The QTL *qBWB02.1* identified in this study was repeatedly detected and explained 12.17–23.33% phenotype variation of BW resistance in three environment conditions. Notably, the cluster of the resistant genes in this region implied that this genome region has potential gene resources for BW disease resistance and worthy further study.

## Conclusion

We used ddRAD-seq technology for large-scale identification of SNPs that were subsequently used for high-throughput genotyping and construction of a high quality genetic map of cultivated peanut. Combining the phenotype data of four environment conditions, we identified four QTLs located on B02 for BW resistance. The major stable QTL region (*qBWB02.1*) covered 2.3 Mb physical distance containing 21 NBS-LRR genes. These genes were considered as putative candidate resistant genes for BW. This study provides a major stable QTL for fine-mapping and contributions to peanut breeding for BW resistance through MAS.

## Data Availability

The datasets generated for this study can be found at https://www.ncbi.nlm.nih.gov/sra/SRP158656.

## Author Contributions

HJ, BL, and JS designed the experiment. LW, XR, and WC planted the mapping population. XZ, LW, and HL performed SNP calling and genotyping. LW, HJ, YC, YL, and LY performed BW resistance evaluation in the mapping population. XZ, WL, LH, and NL performed QTL analysis. LW and XZ wrote the manuscript.

## Conflict of Interest Statement

The authors declare that the researchwas conducted in the absence of any commercial or financial relationships that could be construed as a potential conflict of interest.

## Footnotes

1 http://www.peanutbase.org

## Supplementary Material

TABLE S1 The 2,187 SNPs along with the flanking sequence.

TABLE S2 The lines of the RIL population with *qBWB02.1* region show stable resistance to BW and the lines without *qBWB02.1* region show stable susceptibility to BW.

## References

[B1] Agarwal G. Clevenger J. Pandey M. K. Wang H. Shasidhar Y. Chu Y. (2018). High-density genetic map using whole-genome resequencing for fine mapping and candidate gene discovery for disease resistance in peanut. *Plant Biotechnol. J.* 16 1954–1967. 10.1111/pbi.12930 29637729 PMC6181220

[B2] Baird N. A. Etter P. D. Atwood T. S. Currey M. C. Shiver A. L. Lewis Z. A. (2008). Rapid SNP discovery and genetic mapping using sequenced RAD markers. *PLoS One* 3:e3376. 10.1371/journal.pone.0003376 18852878 PMC2557064

[B3] Ben C. Debellé F. Berges H. Bellec A. Jardinaud M.-F. Anson P. (2013). *Mtqrrs1*, an *R*-locus required for *Medicago truncatula* quantitative resistance to *Ralstonia solanacearum*. *New Phytol.* 199 758–772. 10.1111/nph.12299 23638965

[B4] Bertioli D. J. Cannon S. B. Froenicke L. Huang G. Farmer A. D. Cannon E. K. (2016). The genome sequences of *Arachis duranensis* and *Arachis ipaensis*, the diploid ancestors of cultivated peanut. *Nat. Genet.* 48:438. 10.1038/ng.3517 26901068

[B5] Bhatnagar-Mathur P. Sunkara S. Bhatnagar-Panwar M. Waliyar F. Sharma K. K. (2015). Biotechnological advances for combating *Aspergillus flavus* and aflatoxin contamination in crops. *Plant Sci.* 234 119–132. 10.1016/j.plantsci.2015.02.009 25804815

[B6] Bonardi V. Tang S. Stallmann A. Roberts M. Cherkis K. Dangl J. L. (2011). Expanded functions for a family of plant intracellular immune receptors beyond specific recognition of pathogen effectors. *Proc. Natl. Acad. Sci. U.S.A.* 108 16463–16468. 10.1073/pnas.1113726108 21911370 PMC3182704

[B7] Burow M. D. Simpson C. E. Starr J. L. Paterson A. H. (2001). Transmission genetics of chromatin from a synthetic amphidiploid to cultivated peanut (*Arachis hypogaea* L.). broadening the gene pool of a monophyletic polyploid species. *Genetics* 159 823–827. 11606556 10.1093/genetics/159.2.823 PMC1461827

[B8] Cao B. H. Lei J. J. Wang Y. W. Chen G. J. (2009). Inheritance and identification of scar marker linked to bacterial wilt-resistance in eggplant. *Afr. J. Biotechnol.* 8 5201–5207.

[B9] Carmeille A. Caranta C. Dintinger J. Prior P. Luisetti J. Besse P. (2006). Identification of QTLs for *Ralstonia solanacearum* race 3-phylotype II resistance in tomato. *Theor. Appl. Genet.* 113 110–121. 10.1007/s00122-006-0277-3 16614830

[B10] Catchen J. Hohenlohe P. A. Bassham S. Amores A. Cresko W. A. (2013). Stacks: an analysis tool set for population genomics. *Mol. Ecol.* 22 3124–3140. 10.1111/mec.12354 23701397 PMC3936987

[B11] Catchen J. M. Amores A. Hohenlohe P. Cresko W. Postlethwait J. H. (2011). Stacks: building and genotyping loci de novo from short-read sequences. *G3* 1 171–182. 10.1534/g3.111.000240 22384329 PMC3276136

[B12] Chen B. Y. Jiang H. F. Ren X. P. Liao B. S. Huang J. Q. (2008). Identification and molecular traits of *Arachis* species with resistance to bacterial wilt. *Acta Agric. Boreali Sin.* 23 170–175. 10.1007/s11032-015-0432-0 26869849 PMC4735223

[B13] Chen X. Li X. Zhang B. Xu J. Wu Z. Wang B. (2013). Detection and genotyping of restriction fragment associated polymorphisms in polyploid crops with a pseudo-reference sequence: a case study in allotetraploid *Brassica napus*. *BMC Genomics* 14:346. 10.1186/1471-2164-14-346 23706002 PMC3665465

[B14] Chu Y. Wu C. L. Holbrook C. C. Tillman B. L. Person G. Oziasakins P. (2011). Marker-assisted selection to pyramid nematode resistance and the high oleic trait in peanut. *Plant Genome* 4:110 10.3835/plantgenome2011.01.0001

[B15] Davey J. W. Cezard T. Fuentes-Utrilla P. Eland C. Gharbi K. Blaxter M. L. (2013). Special features of rad sequencing data: implications for genotyping. *Mol. Ecol.* 22 3151–3164. 10.1111/mec.12084 23110438 PMC3712469

[B16] Davey J. W. Hohenlohe P. A. Etter P. D. Boone J. Q. Catchen J. M. Blaxter M. L. (2011). Genome-wide genetic marker discovery and genotyping using next-generation sequencing. *Nat. Rev. Genet.* 12:499. 10.1038/nrg3012 21681211

[B17] Deslandes L. Olivier J. Peeters N. Feng D. X. Khounlotham M. Boucher C. (2003). Physical interaction between RRS1-R, a protein conferring resistance to bacterial wilt, and popp2, a type III effector targeted to the plant nucleus. *Proc. Natl. Acad. Sci. U.S.A.* 100:8024. 10.1073/pnas.1230660100 12788974 PMC164706

[B18] Deslandes L. Olivier J. Theulieres F. Hirsch J. Feng D. X. Bittnereddy P. (2002). Resistance to *Ralstonia solanacearum* in *Arabidopsis thaliana* is conferred by the recessive RRS1-R gene, a member of a novel family of resistance genes. *Proc. Natl. Acad. Sci. U.S.A.* 99 2404–2409. 10.1073/pnas.032485099 11842188 PMC122377

[B19] Foncéka D. Hodo-Abalo T. Rivallan R. Faye I. Sall M. N. Ndoye O. (2009). Genetic mapping of wild introgressions into cultivated peanut: a way toward enlarging the genetic basis of a recent allotetraploid. *BMC Plant Biol.* 9:103. 10.1186/1471-2229-9-103 19650911 PMC3091533

[B20] Fu Y. B. (2014). Genetic diversity analysis of highly incomplete SNP genotype data with imputations: an empirical assessment. *G3* 4 891–900. 10.1534/g3.114.010942 24626289 PMC4025488

[B21] Gautami B. Pandey M. K. Vadez V. Nigam S. N. Ratnakumar P. Krishnamurthy L. (2012). Quantitative trait locus analysis and construction of consensus genetic map for drought tolerance traits based on three recombinant inbred line populations in cultivated groundnut (*Arachis hypogaea* l.). *Mol. Breed.* 30 757–772. 10.1007/s11032-011-9660-0 22924017 PMC3410028

[B22] Godiard L. Sauviac L. Torii K. U. Grenon O. Mangin B. Grimsley N. H. (2003). Erecta, an LRR receptor-like kinase protein controlling development pleiotropically affects resistance to bacterial wilt. *Plant J.* 36 353–365. 10.1046/j.1365-313X.2003.01877.x 14617092

[B23] Grattapaglia D. Sederoff R. (1994). Genetic linkage maps of eucalyptus grandis and eucalyptus urophylla using a pseudo-testcross: mapping strategy and RAPD markers. *Genetics* 137 1121–1137. 7982566 10.1093/genetics/137.4.1121 PMC1206059

[B24] Guo Y. Khanal S. Tang S. Bowers J. E. Heesacker A. F. Khalilian N. (2012). Comparative mapping in intraspecific populations uncovers a high degree of macrosynteny between A- and B-genome diploid species of peanut. *BMC Genomics* 13:608. 10.1186/1471-2164-13-608 23140574 PMC3532320

[B25] Gupta S. K. Baek J. Carrasquilla-Garcia N. Penmetsa R. V. (2015). Genome-wide polymorphism detection in peanut using next-generation restriction-site-associated DNA (RAD) sequencing. *Mol. Breed.* 35:145 10.1007/s11032-015-0343-0

[B26] Hallauer A. R. Miranda J. B. (1998). *Quantitative Genetics in Maize Breeding* 2nd Edn. Ames, IA: Iowa State Univ Press.

[B27] Halward T. Stalker H. T. Kochert G. (1993). Development of an RFLP linkage map in diploid peanut species. *Theor. Appl. Genet.* 87:379. 10.1007/BF01184927 24190266

[B28] Herselman L. Thwaites R. Kimmins F. M. Courtois B. Merwe P. J. Seal S. E. (2004). Identification and mapping of AFLP markers linked to peanut (*Arachis hypogaea* L.) resistance to the aphid vector of groundnut rosette disease. *Theor. Appl. Genet.* 109 1426–1433. 10.1007/s00122-004-1756-z 15290049

[B29] Hong Y. Chen X. Liang X. Liu H. Zhou G. Li S. (2010). A SSR-based composite genetic linkage map for the cultivated peanut (*Arachis hypogaea* L.) genome. *BMC Plant Biol.* 10:17. 10.1186/1471-2229-10-17 20105299 PMC2835713

[B30] Hong Y. B. Wen S. J. Zhong N. Xing-Yu L. I. Zhu F. H. Liang X. Q. (2011). Correlation between SSR markers and resistance to bacterial wilt and rust in peanut. *Guangdong Agric. Sci.* S1 61–63. 10.16768/j.issn.1004-874x.2011.s1.019

[B31] Janila P. Variath M. T. Pandey M. K. Desmae H. Motagi B. N. Okori P. (2016). Genomic tools in groundnut breeding program: status and perspectives. *Front. Plant Sci.* 7:289. 10.3389/fpls.2016.00289 27014312 PMC4794498

[B32] Jiang H. F. Chen B. Y. Ren X. P. Liao B. S. Lei Y. Fu T. D. (2007). Identification of SSR markers linked to bacterial wilt resistance of peanut with RILs. *Chin. J. Oil Crop Sci.* 29 26–30.

[B33] Jiang H. F. Duan N. X. Ren X. P. (2006). *Descriptors and Data Standard for Peanut (Arachs spp.)* 1st Edn. Beijing: China Agric. Press 59–77.

[B34] Liao B.-S. Lei Y. Li D. Wang S.-Y. Huang J.-Q. Ren X.-P. (2010). Novel high oil germplasm with resistance to *Aspergillus flavus* and bacterial wilt developed from recombinant inbred lines. *Acta Agronomica Sinica* 36 1296–1301. 10.3724/SP.J.1006.2010.01296

[B35] Lyu J. Jiang H. Ren X. Zhang X. Liao B. (2010). Identification and molecular traits of ICRISAT mini core collection peanut species with resistance to bacterial wilt. *Chin. Agric. Sci. Bull.* 26 47–51.

[B36] McCouch S. R. Cho Y. G Yano M. Paul E. Blinstrub M. Morishima H. (1997). Report on QTL nomenclature. *Rice Genet. Newsl.* 14 11–131. 23813016

[B37] Nagy E. D. Guo Y. Tang S. Bowers J. E. Okashah R. A. Taylor C. A. (2012). A high-density genetic map of *Arachis duranensis*, a diploid ancestor of cultivated peanut. *BMC Genomics* 13:469. 10.1186/1471-2164-13-469 22967170 PMC3542255

[B38] Narusaka M. Hatakeyama K. Shirasu K. Narusaka Y. (2014). *Arabidopsis* dual resistance proteins, both RPS4 and RRS1, are required for resistance to bacterial wilt in transgenic *Brassica* crops. *Plant Signal. Behav.* 9:e29130. 10.4161/psb.29130 25763492 PMC4203570

[B39] Peng W. F. Jiang H. F. Ren X. P. Jian-Wei L. U. Zhao X. Y. Huang L. (2010). Construction of AFLP genetic linkage map and detection of QTLs for bacterial wilt resistance in peanut (*Arachis hypogaea* L.). *Acta Agric. Boreali Sin.* 25 81–86.

[B40] Peterson B. K. Weber J. N. Kay E. H. Fisher H. S. Hoekstra H. E. (2012). Double digest radseq: an inexpensive method for de novo snp discovery and genotyping in model and non-model species. *PLoS One* 7:e37135. 10.1371/journal.pone.0037135 22675423 PMC3365034

[B41] Prasath D. El-Sharkawy I. Sherif S. Tiwary K. S. Jayasankar S. (2011). Cloning and characterization of pr5 gene from *Curcuma amada* and *Zingiber officinalein* response to *Ralstonia solanacearum* infection. *Plant Cell Rep.* 30 1799–1809. 10.1007/s00299-011-1087-x 21594675

[B42] Qian Y. Wang X. Wang D. Zhang L. Zu C. Gao Z. (2013). The detection of QTLs controlling bacterial wilt resistance in tobacco (*N. tabacum* L.). *Euphytica* 192 259–266. 10.1007/s10681-012-0846-2

[B43] Qin H. Feng S. Chen C. Guo Y. Knapp S. Culbreath A. (2012). An integrated genetic linkage map of cultivated peanut (*Arachis hypogaea* L.) constructed from two RIL populations. *Theor. Appl. Genet.* 124 653–664. 10.1007/s00122-011-1737-y 22072100

[B44] Recknagel H. Elmer K. R. Meyer A. (2013). A hybrid genetic linkage map of two ecologically and morphologically divergent Midas cichlid fishes (*Amphilophus* spp.) obtained by massively parallel DNA sequencing (ddRADseq). *G3 Genes Genet.* 3:65. 10.1534/g3.112.003897 23316439 PMC3538344

[B45] Ren X. P. Jiang H. F. Liao B. S. (2008). Identification of molecular markers for resistance to bacterial wilt in peanut (*Arachis hypogaea* L.). *J. Plant Genet. Res.* 9 163–167. 26869849

[B46] Seck K. Samb N. Tempesta S. Mulanga-Kabeya C. Henzel D. Sow P. S. (2013). Integrated consensus map of cultivated peanut and wild relatives reveals structures of the A and B genomes of Arachis and divergence of the legume genomes. *DNA Res.* 20:173. 10.1093/dnares/dss042 23315685 PMC3628447

[B47] Shasidhar Y. Vishwakarma M. K. Pandey M. K. Janila P. Variath M. T. Manohar S. S. (2017). Molecular mapping of oil content and fatty acids using dense genetic maps in groundnut (*Arachis hypogaea* L.). *Front Plant Sci.* 8:794. 10.3389/fpls.2017.00794 28588591 PMC5438992

[B48] Shi J. R. Xu D. H. Yang H. Y. Lu Q. X. Ban T. (2008). DNA marker analysis for pyramided of *Fusarium* head blight (FHB) resistance QTLs from different germplasm. *Genetica* 133 77–84. 10.1007/s10709-007-9186-x 17676412

[B49] Shirasawa K. Bertioli D. J. Varshney R. K. Moretzsohn M. C. Lealbertioli S. C. M. Thudi M. (2013). Integrated consensus map of cultivated peanut and wild relatives reveals structures of the A and B genomes of *Arachis* and divergence of the legume genomes. *DNA Res.* 20 173–184. 10.1093/dnares/dss042 23315685 PMC3628447

[B50] Shirasawa K. Koilkonda P. Aoki K. Hirakawa H. Tabata S. Watanabe M. (2012). In silico polymorphism analysis for the development of simple sequence repeat and transposon markers and construction of linkage map in cultivated peanut. *BMC Plant Biol.* 12:80. 10.1186/1471-2229-12-80 22672714 PMC3404960

[B51] Sujay V. Gowda M. V. C. Pandey M. K. Bhat R. S. Khedikar Y. P. Nadaf H. L. (2012). Quantitative trait locus analysis and construction of consensus genetic map for foliar disease resistance based on two recombinant inbred line populations in cultivated groundnut (*Arachis hypogaea* l.). *Mol. Breed.* 30 773–788. 10.1007/s11032-011-9661-z 22924018 PMC3410029

[B52] Sukruth M. Paratwagh S. A. Sujay V. Kumari V. Gowda M. V. C. Nadaf H. L. (2015). Validation of markers linked to late leaf spot and rust resistance, and selection of superior genotypes among diverse recombinant inbred lines and backcross lines in peanut (*Arachis hypogaea* L.). *Euphytica* 204 1–9. 10.1007/s10681-014-1339-2

[B53] Sunkara S. Bhatnagar-Mathur P. Sharma K. K. (2014). “Transgenic interventions in peanut crop improvement: progress and prospects,” in *Genetics, Genomics and Breeding of Peanuts* eds Mallikarjuna N. Varshney R. K. (Boca Raton, FL: CRC press) 179–216.

[B54] Van Ooijen J. W. (2006). *JoinMap 4 Software for The Calculation of Genetic Linkage Maps in Experimental Populations.* Wageningen: Kyazma.

[B55] Varshney R. K. Bertioli D. J. Moretzsohn M. C. Vadez V. Krishnamurthy L. Aruna R. (2009). The first SSR-based genetic linkage map for cultivated groundnut (*Arachis hypogaea* L.). *Theor. Appl. Genet.* 118 729–739. 10.1007/s00122-008-0933-x 19048225

[B56] Varshney R. K. Graner A. Sorrells M. E. (2005). Genomics-assisted breeding for crop improvement. *Trends Plant Sci.* 10 621–630. 10.1016/j.tplants.2005.10.004 16290213

[B57] Varshney R. K. Hoisington D. A. Tyagi A. K. (2006). Advances in cereal genomics and applications in crop breeding. *Trends Biotech.* 24 490–499. 10.1016/j.tibtech.2006.08.006 16956681

[B58] Varshney R. K. Pandey M. K. Janila P. Nigam S. N. Sudini H. Gowda M. V. C. (2014a). Marker-assisted introgression of a qtl region to improve rust resistance in three elite and popular varieties of peanut (*Arachis hypogaea* L.). *Theor. Appl. Genet.* 127 1771–1781. 10.1007/s00122-014-2338-3 24927821 PMC4110420

[B59] Varshney R. K. Thudi M. Nayak S. N. Gaur P. M. Kashiwagi J. Krishnamurthy L. (2014b). Genetic dissection of drought tolerance in chickpea (*Cicer arietinum* L.). *Theor. Appl. Genet.* 127 445–462. 10.1007/s00122-013-2230-2236 24326458 PMC3910274

[B60] Varshney R. K. Saxena R. K. Upadhyaya H. D. Khan A. W. Yu Y. Kim C. (2017). Whole-genome resequencing of 292 pigeonpea accessions identifies genomic regions associated with domestication and agronomic traits. *Nat. Genet.* 49 1082–1088. 10.1038/ng.3872 28530677

[B61] Varshney R. K. Thundi M. May G. D. Jackson S. A. (2010). “Legume genomics and breeding,” in *Plant Breeding Review* ed. Janick J. (Hoboken, NJ: John Wiley and Sons, Inc). 10.1002/9780470535486.ch6

[B62] Voorrips R. E. (2002). MapChart: software for the graphical presentation of linkage maps and QTLs. *J. Hered.* 93 77–78. 10.1093/jhered/93.1.77 12011185

[B63] Wang H. Penmetsa R. V. Yuan M. Gong L. Zhao Y. Guo B. (2012). Development and characterization of BAC-end sequence derived SSRs, and their incorporation into a new higher density genetic map for cultivated peanut (*Arachis hypogaea* L.). *BMC Plant Biol.* 12:10. 10.1186/1471-2229-12-10 22260238 PMC3298471

[B64] Wang J. F. Olivier J. Thoquet P. Mangin B. Sauviac L. Grimsley N. H. (2000). Resistance of tomato line Hawaii7996 to *Ralstonia solanacearum* Pss4 in Taiwan is controlled mainly by a major strain-specific locus. *Mol. Plant Microbe. Interact.* 13 6–13. 10.1094/MPMI.2000.13.1.6 10656580

[B65] Wang S. Basten C. J. Zeng Z. B. (2012). *Windows QTL Cartographer 2.5.* Raleigh, NC: Department of Statistics, North Carolina State University.

[B66] Wicker E. Grassart L. Coranson-Beaudu R. Mian D. Guilbaud C. Fegan M. (2007). *Ralstonia solanacearum* strains from martinique (French west indies) exhibiting a new pathogenic potential. *Appl. Environ. Microbiol.* 73 6790–6801. 10.1128/AEM.00841-07 17720825 PMC2074947

[B67] Xiao X. Cao B. Li G. Lei J. Chen Q. Jin J. (2015). Functional characterization of a putative bacterial wilt resistance gene (*RE-bw*) in eggplant. *Plant Mol. Biol. Rep.* 33:1058 10.1007/s11105-014-0814-1

[B68] Xiong W. X. Yuan J. Z. Yu H. Yu C. Q. Xiong R. F. (2003). New cultivation techniques for new variety peanut Yuanza 9102. *Peanut Sci.* 32(Suppl. 1) 500–503.

[B69] Yu S. L. Wang C. T. Yang Q. T. Zhang D. X. Zhang X. Y. Cao Y. L. (2011). *Peanut Genetics and Breeding in China, 565.* Shanghai: Shanghai Scientific and Technology Press.

[B70] Zhang C. Chen H. Cai T. Deng Y. Zhuang R. Zhang N. (2017). Overexpression of a novel peanut NBS-LRR gene AhRRS5 enhances disease resistance to *Ralstonia solanacearum*in tobacco. *Plant Biotechnol. J.* 15 39–55. 10.1111/pbi.12589 27311738 PMC5253469

[B71] Zhao Y. Zhang C. Chen H. Yuan M. Nipper R. Prakash C. S. (2016). QTL mapping for bacterial wilt resistance in peanut ( *Arachis hypogaea* L.). *Mol. Breed.* 36:13. 10.1007/s11032-015-0432-0 26869849 PMC4735223

[B72] Zhou X. Xia Y. Ren X. Chen Y. Li H. Huang S. (2014). Construction of a SNP-based genetic linkage map in cultivated peanut based on large scale marker development using next-generation double-digest restriction-site-associated DNA sequencing (ddRADseq). *BMC Genomics* 15:351. 10.1186/1471-2164-15-351 24885639 PMC4035077

